# Autoantibodies in juvenile-onset myositis: Their diagnostic value and associated clinical phenotype in a large UK cohort

**DOI:** 10.1016/j.jaut.2017.06.007

**Published:** 2017-11

**Authors:** Sarah L. Tansley, Stefania Simou, Gavin Shaddick, Zoe E. Betteridge, Beverley Almeida, Harsha Gunawardena, Wendy Thomson, Michael W. Beresford, Angela Midgley, Francesco Muntoni, Lucy R. Wedderburn, Neil J. McHugh

**Affiliations:** a Royal National Hospital for Rheumatic Diseases, Royal United Hospitals Bath NHS Foundation Trust, Upper Borough Walls, Bath, BA1 1RL, UK; b Department of Pharmacy and Pharmacology, University of Bath, Claverton Down, Bath, BA2 7AY, UK; c Inflammation and Rheumatology Section, UCL Institute of Child Health and Arthritis Research UK Centre for Adolescent Rheumatology at UCL, UCLH and GOSH, London, UK; d Department of Mathematics, University of Bath, Claverton Down, Bath, BA2 7AY, UK; e Department of Rheumatology, Great Ormond Street Children's Hospital, London, UK; f Clinical and Academic Rheumatology, North Bristol NHS Trust, Southmead Hospital, Bristol, BS10 5NB, UK; g Arthritis Research UK Centre Genetics and Genomics, Faculty of Biology, Medicine and Health, Manchester Academic Health Science Centre, The University of Manchester and NIHR Manchester Musculoskeletal Biomedical Research Unit, Central Manchester NHS Foundation Trust, UK; h Institute of Translational Medicine, University of Liverpool, Liverpool, UK; i Department of Paediatric Rheumatology, Alder Hey Children's NHS Foundation Trust, Liverpool, UK; j Arthritis Research UK Experimental Arthritis Treatment Centre for Children, Liverpool, UK; k Developmental Neuroscience Programme, UCL Great Ormond Street Institute of Child Health, MRC Centre for Neuromuscular Diseases, UCL, London, UK; l Arthritis Research UK Centre for Adolescent Rheumatology at UCL, UCLH and GOSH, London, UK

**Keywords:** Myositis, Paediatric rheumatology, Autoantibody, Phenotype, Autoimmune disease, Myopathy

## Abstract

**Objectives:**

Juvenile myositis is a rare and heterogeneous disease. Diagnosis is often difficult but early treatment is important in reducing the risk of associated morbidity and poor outcomes. Myositis specific autoantibodies have been described in both juvenile and adult patients with myositis and can be helpful in dividing patients into clinically homogenous groups. We aimed to explore the utility of myositis specific autoantibodies as diagnostic and prognostic biomarkers in patients with juvenile-onset disease.

**Methods:**

Using radio-labelled immunoprecipitation and previously validated ELISAs we examined the presence of myositis specific autoantibodies in 380 patients with juvenile-onset myositis in addition to, 318 patients with juvenile idiopathic arthritis, 21 patients with juvenile-onset SLE, 27 patients with muscular dystrophies, and 48 healthy children.

**Results:**

An autoantibody was identified in 60% of juvenile-onset myositis patients. Myositis specific autoantibodies (49% patients) were exclusively found in patients with myositis and with the exception of one case were mutually exclusive and not found in conjunction with another autoantibody. Autoantibody subtypes were associated with age at disease onset, key clinical disease features and treatment received.

**Conclusions:**

In juvenile patients the identification of a myositis specific autoantibody is highly suggestive of myositis. Autoantibodies can be identified in the majority of affected children and provide useful prognostic information. There is evidence of a differential treatment approach and patients with anti-TIF1γ autoantibodies are significantly more likely to receive aggressive treatment with IV cyclophosphamide and/or biologic drugs, clear trends are also visible in other autoantibody subgroups.

## Highlights

• Using immunoprecipitation, an autoantibody is identifiable in most children with juvenile-onset myositis.
• Myositis specific autoantibodies are highly specific and were not identified in control groups.
• Autoantibodies identify patient subgroups associated with clinically important disease features.
• Evidence of an existing differential treatment approach between autoantibody subgroups is presented.

## Abbreviations

JDCBS: Juvenile Dermatomyositis Cohort and Biomarker Study
CAPS: Childhood Arthritis Prospective Cohort Study
CMAS: Childhood Myositis Assessment Score
PGA: Physician Global Assessment visual analogue score
Anti-TIF1γ: anti-transcription intermediary factor gamma autoantibody
Anti-NXP2: anti-nuclear matrix protein 2 autoantibody
Anti-MDA5: anti-melanoma differentiation associated protein 5 autoantibody
Anti-SRP: anti-signal recognition peptide autoantibody
Anti-HMGCR: anti-3-hydroxy-3-methyl-glutaryl-coenzyme A reductase autoantibody
Anti-SAE: anti-small ubiquitin-like modifier activating enzyme autoantibody
Anti-PmScl: anti-Polymyositis Scleroderma autoantibody
Anti-U1RNP: anti-U1 Ribonucleoprotein autoantibody
MSA: Myositis specific autoantibody
MAA: Myositis associated autoantibody

## 1 Introduction

Juvenile-onset myositis refers to a group of rare childhood autoimmune diseases that typically present with proximal muscle weakness and elevated muscle enzymes; more than 90% of affected children have associated skin disease and are thus classified as Juvenile Dermatomyositis (JDM) [1]. Juvenile myositis is clinically highly heterogeneous with muscle weakness ranging from profound and requiring hospitalisation, to clinically amyopathic dermatomyositis with normal muscle strength. Extra-muscular disease including skin and internal organ involvement contributes significantly to disease morbidity. Patient sub-stratification is desirable to inform prognosis and guide further investigation and treatment. Traditionally subgroups based on clinical and histopathological criteria include polymyositis, dermatomyositis and overlap syndromes but this classification fails to explain all of the variation in what is a complex disease and the boundaries between traditional subgroups are becoming increasingly indistinct. Autoantibodies identifiable in patients with myositis are often described as either myositis specific (MSA) or myositis associated (MAA). MSA are believed to occur exclusively in patients with an idiopathic inflammatory myopathy while MAA may also occur in patients with other connective tissue diseases or an overlap disorder. Collectively autoantibodies have been identified in 60–70% of patients with juvenile myositis and can divide patients into clinically homogenous subgroups [2], [3], [4], [5], [6]. There is growing evidence for the utility of autoantibodies as biomarkers to predict disease features and outcome in juvenile myositis [2], [3], [4], [5], [6], [7], [8].

Despite well described pathognomonic features, the diagnosis of juvenile myositis can be challenging; a recent study from North America reported a median delay in diagnosis of 4–6 months [9]. The differential diagnosis of muscular weakness in children is wide and additional features such as arthralgia or Raynaud's phenomenon may lead to consideration of other more common childhood rheumatological diseases such as juvenile idiopathic arthritis (JIA) or juvenile-onset systemic lupus erythematosus (JSLE). The possibility of overlap disorders compounds this problem. Furthermore, the muscular dystrophies and other genetic muscle diseases are important to exclude. It is crucial that diagnostic difficulties can be overcome as early diagnosis and initiation of aggressive treatment has been shown to reduce morbidity and improve patient outcome [9], [10], [11], [12]. Myositis specific autoantibodies are believed to occur exclusively in patients with myositis and have not been found in patients with genetic muscle disease in the absence of a coexistent inflammatory myopathy [13]. As standard testing for myositis specific autoantibodies becomes more widely available, there is growing interest in their use in diagnosis and predicting prognosis. In this study we analyse the prevalence and clinical associations of MSA/MAA in a large cohort of UK children with juvenile myositis compared to healthy children and those suffering from diseases with overlapping clinical features, JIA and JSLE.

## 2 Materials and methods

### 2.1 Patients with juvenile myositis

Patient serum samples and clinical data were available for 380 children enrolled in the UK Juvenile Dermatomyositis Cohort and Biomarker Study (JDCBS). The JDCBS is a large cohort of UK patients with myositis, the majority with JDM [1]. Patients are recruited from paediatric rheumatology departments across the UK, and data are collected prospectively on standardised proformas. Patients aged ≤16 years are included based on a diagnosis of definite or probable JDM or polymyositis by Bohan and Peter criteria [14]; as well as JDM or polymyositis with overlap connective tissue disease features. The JDCBS was established in 2001 and many patients have more than 15 years of follow-up data available. The median length of time from symptom onset to time of analysis of patients included in this study was 9.31 years.

We investigated the presence or absence of key disease features occurring at any point over the follow-up period including calcinosis, dysphagia, cutaneous ulceration, lipoatrophy and arthritis. The lowest ever recorded childhood myositis assessment score (CMAS) was used as a measure of the maximum recorded muscle weakness: CMAS is a systematic and validated measure of muscle strength in children with juvenile myositis. The score ranges between 0 and 52, with lower scores corresponding to a greater degree of clinical weakness [15]. We used the highest ever recorded physician global assessment visual analogue score (PGA) as a proxy measure for maximal disease activity/severity. PGA graded 0–10, is used as an overall measure of disease activity, a higher score reflecting more active disease.

In the UK first line treatment for juvenile-onset myositis is typically methotrexate with cortico-steroids, a regime recently been shown in an international randomised trial, to be optimal compared to steroids alone [16]. Strict guidelines exist for the administration of biologic drugs and these are reserved for the most unwell patients, who have failed to respond to first-line medications. We determined whether patients had at any point received treatment with any biologic drug and/or intravenous cyclophosphamide.

### 2.2 Patients with JIA

Patient serum samples were obtained for 318 children enrolled in the Childhood Arthritis Prospective Study (CAPS), a prospective longitudinal inception cohort study of children with new onset inflammatory arthritis [17]. Patients are recruited from 7 tertiary referral centres across the UK. Children aged ≤16 years with newly diagnosed inflammatory arthritis in one or more joints, which had persisted for at least 2 weeks, are invited to participate. Exclusion criteria include septic arthritis, haemarthrosis, arthritis caused by malignancy or trauma and connective tissue disease.

### 2.3 Patients with JSLE

Patient serum samples were obtained for 21 children enrolled in the UK Juvenile Systemic Lupus Erythematosus (JSLE) Cohort Study and Repository. Patients with definite or probable JSLE diagnosed aged ≤16 years are recruited from centres across the UK. All JSLE samples included here were collected from patients being cared for in the Department of Paediatric Rheumatology, Alder Hey Children's NHS Foundation Trust. The data collection and repository has previously been described [18].

### 2.4 Muscular dystrophy

Patient serum samples were obtained from 27 children with muscular dystrophies (20 Duchene muscular dystrophy, 5 Becker muscular dystrophy and 2 Limb girdle type 2D muscular dystrophy) through the Medical Research Centre for Neuromuscular Disorders Biobank at University College London.

### 2.5 Healthy controls

Serum was obtained from 48 healthy subjects aged ≤16 years attending Alder Hey Children's NHS Foundation Trust, Liverpool, UK for elective surgery where no intercurrent infection or family history of autoimmune disease was present, as part of the UK JSLE Cohort Study (see above).

For all studies ethical approval has been obtained and parental consent for children, and consent or age-appropriate assent was obtained for all patients in accordance with the declaration of Helsinki.

### 2.6 Myositis specific autoantibody detection

Immunoprecipitation of radiolabelled K562 cells was performed on all samples to determine the presence of autoantibodies as previously described [2], [3], [5], [19]. Ten microlitres of sera were mixed with 2 mg of protein A Sepharose beads (Sigma, St Louis, MO, USA) in immunoprecipitation (IPP) buffer (10 mM Tris-HCl, pH 8.0, 500 mM NaCl, 0.1% v/v Igepal) at room temperature for 30 min. Beads were washed in IPP buffer prior to the addition of 120 ml of [35S] methionine-labelled K562 cell extract in IPP buffer. Samples were mixed at 4 °C for 2 h. Beads were washed in IPP buffer and Tris-buffered saline (10 mM Tris-HCl, pH 7.4, 150 mM NaCl) before being re-suspended in 50 ml of SDS sample buffer (Sigma). After heating, proteins were fractionated by SDS-PAGE, enhanced, fixed and dried. Labelled proteins were analysed by autoradiography. Sera known to contain the following autoantibodies were always included as controls; normal serum, anti-Jo-1, anti-U1RNP, anti-RNAPII, anti-PmScl, anti- Ro 60, anti-La, anti-Mi2, anti-Ku, anti-SAE anti-RNAPI/III, anti-U3RNP anti-p155/140, anti-PL7, anti-PL12, anti- Zo, anti-SRP, anti-Scl-70, and anti-NXP2.

**Fig. 1:**
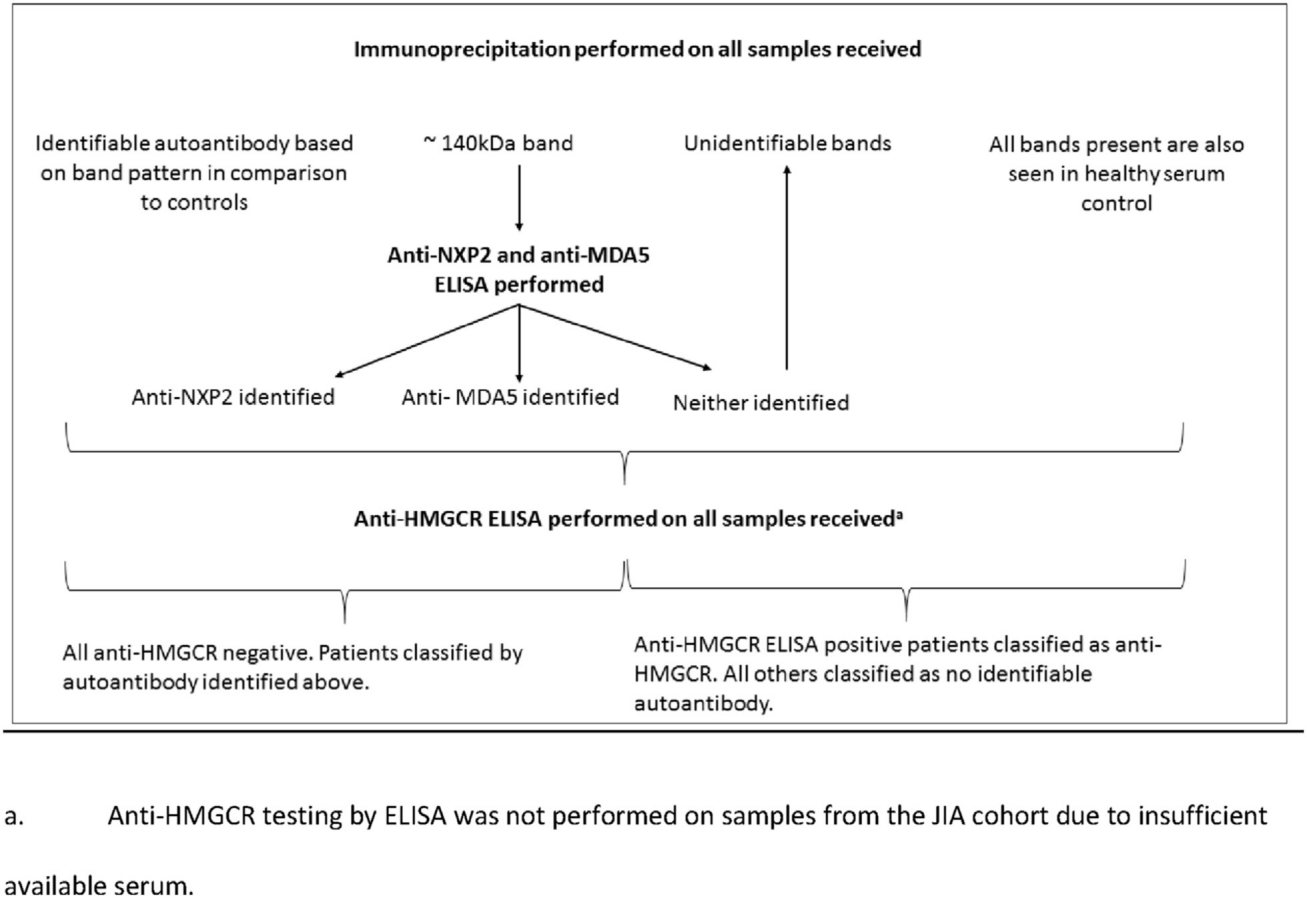
A flow chart describing the autoantibody detection process. a. Anti-HMGCR testing by ELISA was not performed on samples from the JIA cohort due to insufficient available serum.

As immunoprecipitation is unable to identify all myositis specific and associated autoantibodies of interest additional testing was performed as follows: In patients with a 140 kDa band on immunoprecipitation specificity for anti-NXP2 or anti-MDA5 was determined by ELISA as previously published [2], [3]. Where small nuclear ribonuclear proteins were seen on immunoprecipitation the presence of anti-U1RNP and/or anti-Sm was determined by Western blotting of a Hep2 cell extract as previously described [20]. With the exception of the JIA patient cohort, where insufficient serum was available, the presence of anti-HMGCR was determined in all patient and control samples by ELISA using recombinant antigen, as previously described [21]. See Fig. 1 for an overview of the autoantibody detection process.

### 2.7 Statistical analysis

Statistical analysis was performed using R [22]. For independent analyses, chi-squared tests were used to assess potential differences between groups e.g. autoantibody prevalence in patients classified as PM or not PM. Generalised linear models were used to determine the relationship between key disease features and autoantibody subgroups using logistic regression for binary variables and Poisson regression for categories of continuous variables. In this way the clinical features of patients within an autoantibody defined subgroup were compared to the remainder of the cohort allowing adjustment for variables including other autoantibody subgroups, age at disease onset and disease duration. The selection of variables included in the final models was made based on Akaike information criterion by considering all possible subsets of variables [23].

## 3 Results

### 3.1 Myositis specific autoantibodies are exclusively found in those patients with myositis

Demographic data for the different cohorts are shown in Table 1. Myositis specific or associated autoantibodies were identified in 225 (59%) of patients in the myositis cohort but in none of the patients of the other disease groups (318 JIA, 21 JSLE, 27 muscular dystrophy) or healthy children, with the exception of the MAA anti-U1RNP which was found in 38% of JSLE patients. One JDM patient was found to have more than one autoantibody; anti-TIF1γ and anti-U1RNP and this case was excluded from further phenotype analyses. The myositis cohort contained 8 patients identified as ‘other idiopathic inflammatory myopathy’ comprising patients with focal myositis, viral myositis and CANDLE syndrome; it is noteworthy that none of this group had a detectable autoantibody.

**Table 1:** Demographic and autoantibody data for the 793 children included in this study.

Cohort (n)	Median age at onset (IQR)	Female n (%)	Autoantibody identifiable (%)		
			Total	MSA	MAA
Juvenile myositis (379)^a^	6.8 (3.9–10.1)	267^a^ (70)	225 (59)	185 (49)	40 (11)
JDM (316)	6.3 (3.7–10.0)	218 (69)	187 (59)	174 (55)	13 (4)
JPM (6)	12.0 (11.4–13.5)	4 (67)	6 (100)	5 (83)	1 (17)
Overlap CTD or MCTD (49)	9.0 (6.7–11.7)	40 (83)	31 (63)	6 (12)	25 (51)
Other IIM^b^ (8)	8.9 (3.1–12.8)	7 (58)	0	0	0
JIA (318^c^)	6.3 (2.8–10.4)	203 (63)	0	0	0
JSLE (21)	16.3 (14.4–16.7)	17 (81)	8 (38)	0	8 (38)^d^
Muscular dystrophy^e^ (27)	9 (7.5–14)	2 (7.4)	0	0	0
Healthy controls (48)	13.4 (10.9–14.8)	25 (52)	0	0	0

IQR; interquartile range MSA; myositis specific autoantibody MAA; myositis associated autoantibody JDM′ juvenile dermatomyositis JPM; juvenile polymyositis CTD; connective tissue disease MCTD; mixed connective tissue disease JIA; juvenile idiopathic arthritis JSLE; juvenile-onset systemic lupus erythematosus.

a 380 patients screened and one patient with anti-TIF1γ and anti-U1RNP excluded from further analysis.

b 2 focal myositis, 2 brothers with CANDLE syndrome, 2 viral/post-infective myositis, 1 C1q deficiency infantile lupus, 1 ‘inflammatory myopathy and panniculitis’.

c 16 systemic, 164 oligoarticular, 8 rheumatoid factor positive polyarthritis, 78 rheumatoid factor negative polyarthritis, 22 psoriatic arthritis, 15 enthesitis related arthritis, 7 undifferentiated, 8 missing data.

d All anti-U1RNP.

e 20 Duchenne muscular dystrophy, 5 Becker muscular dystrophy and 2 Limb Girdle muscle dystrophy type 2D.

Some situations can make the diagnosis of juvenile myositis more challenging. Ten patients (2.6%) were classified polymyositis or polymyositis with overlap Connective Tissue Disease (CTD), and did not have a rash at presentation. A further 89 (25%) had a lowest ever recorded CMAS ≥48, corresponding to no clinically detectable weakness [24]. In both groups the prevalence of an autoantibody was 80% and 54% respectively and not significantly different from the remainder of the cohort (p = 0.33 and 0.27 respectively), emphasising that analysis for autoantibodies may be highly informative in these diagnostically challenging cases.

### 3.2 Myositis specific and associated autoantibody prevalence

**Fig. 2:**
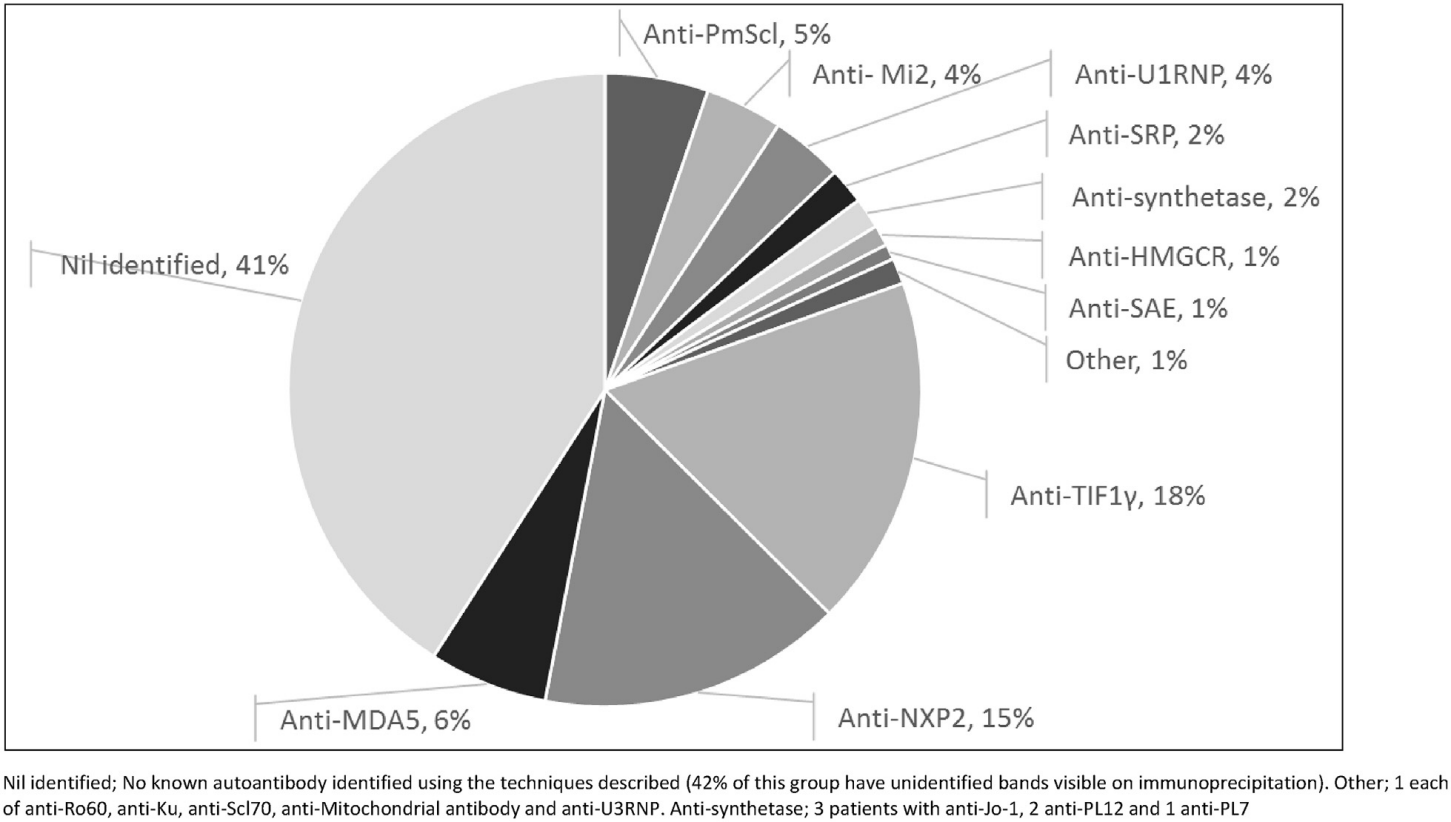
The prevalence of myositis specific and associated autoantibodies in the juvenile-onset myositis cohort (n = 379). An autoantibody was identified in 225 patients (59%). The most common autoantibody subgroups were anti-TIF1γ (18%), anti-NXP2 (15%) and anti-MDA5 (6%). Alternative autoantibodies were collectively identified in the remaining 20%. Nil identified; No known autoantibody identified using the techniques described (42% of this group have unidentified bands visible on immunoprecipitation). Other; 1 each of anti-Ro60, anti-Ku, anti-Scl70, anti-Mitochondrial antibody and anti-U3RNP. Anti-synthetase; 3 patients with anti-Jo-1, 2 anti-PL12 and 1 anti-PL7.

**Fig. 3:**
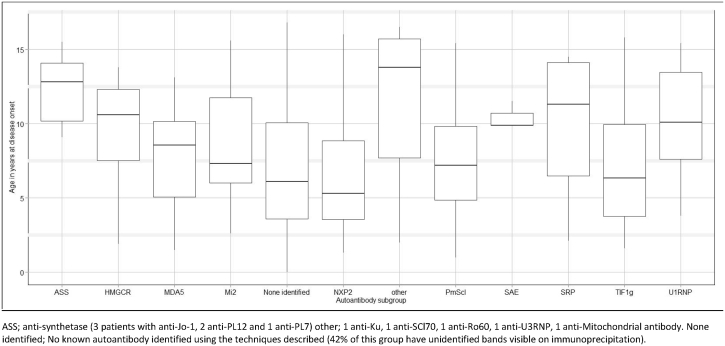
The median age at disease onset for patients with juvenile-onset myositis was 6.9 years. As illustrated, the median age at disease onset varied between autoantibody subgroups. Anti-U1RNP, anti-synthetase and ‘other’ myositis associated autoantibodies were all more likely to be identified in older patients. ASS; anti-synthetase (3 patients with anti-Jo-1, 2 anti-PL12 and 1 anti-PL7) other; 1 anti-Ku, 1 anti-SCl70, 1 anti-Ro60, 1 anti-U3RNP, 1 anti-Mitochondrial antibody. None identified; No known autoantibody identified using the techniques described (42% of this group have unidentified bands visible on immunoprecipitation).

The prevalence of autoantibody subgroups in the juvenile myositis cohort is shown in Fig. 2. Autoantibody prevalence was influenced by age at disease onset, see below and Fig. 3. Patients with anti-HMGCR were more likely to describe themselves as ‘other’ ethnicity (OR 2.78 (1.26–6.15), p = 0.011), (options included White, Black, Indian subcontinent and other), but no other associations between autoantibody and ethnic background were identified.

### 3.3 Autoantibody associated disease features

Using generalised linear regression analyses, as described above, we investigated associations between key muscular and extra-muscular disease features and autoantibody subgroups. Table 2A summaries the disease phenotype associated with each autoantibody in terms of key clinical features. Table 2B summarises the disease severity of autoantibody associated disease groups in terms of muscle strength, physician global assessment score and the likelihood of receiving second-line therapies. Only two deaths are known to have occurred in the JDCBS cohort as a whole and no patient has been reported to have presented with or subsequently developed a malignancy, as such mortality and malignancy were not analysed further in conjunction with autoantibody status.

**Table 2A:** The association of key clinical disease features with autoantibody subgroups.

	Classified as PM (%)	Dysphagia^a^ (%)	Calcinosis^a^ (%)	Ulceration^a^ (%)	Oedema^a^ (%)	Lipoatrophy^a^ (%)	Arthritis^a^ (%)
Total cohort (n = 379)^b^	10 (2.6)	96 (26)	120 (32)	76 (20)	155 (41)	57 (15)	148 (40)
Anti-TIF1γ (n = 68)	0	18 (26)	25 (37)	23 (34)***	32 (47)	13 (19)	26 (38)
Anti-NXP2 (n = 59)	0	17 (29)	25 (43)	8 (14)	26 (44)	9 (16)	20 (34)
Anti-MDA5 (n = 23)	0	7 (30)	7 (27)	11 (50)***	10 (43)	3 (13)	15 (65)*
Anti-PmScl (n = 20)	0	5 (25)	11 (55)*	4 (20)	5 (25)	6 (30)*	11 (44)
Anti- Mi2 (n = 15)	0	7 (47)*	4 (27)	0	11 (73)*	2 (13)	6 (40)
Anti-U1RNP (n = 14)	3 (21)***	6 (43)	1 (8)	2 (14)	2 (14)	2 (14)	7 (50)
Anti-SRP (n = 7)	2 (29)***	3 (43)	1 (14)	4 (50)**	5 (71)	1 (13)	2 (29)
Anti-synthetase^c^ (n = 6)	0	0	1 (17)	2 (33)	3 (50)	2 (33)*	2 (33)
Anti-HMGCR (n = 4)	2 (50)***	3 (75)*	0	0	0	0	0
Anti-SAE (n = 3)	0	0	1 (33)	1 (33)	1 (33)	1 (33)	2 (66)
Other^d^ (n = 5)	1 (20)**	3 (60)	1 (20)	2 (50)	1 (20)	2 (50)	3 (60)
Nil identified^e^ (n = 155)	2 (1)	27 (17)	44 (28)	19 (12)	59 (38)	16 (10)	54 (35)

Statistically significant associations are highlighted in bold.

*P < 0.05, **P < 0.01, ***P < 0.001, PM; polymyositis.

a Data available for 374 patients.

b Including 8 patients with other idiopathic inflammatory myopathy.

c 3 patients anti-Jo-1, 2 patients anti-PL12 and 1 patient anti-PL7.

d 1 anti-Ku, 1 anti-Scl70, 1 anti-Ro60, 1 anti-U3RNP, 1 anti-mitochondrial antibody.

e No known autoantibody identified using the techniques described (42% of this group have unidentified bands visible on immunoprecipitation).

**Table 2B:** The association of key clinical outcome measures with autoantibody subgroups.

	Lowest ever CMAS^a^ median (IQR)	Highest ever PGA^b^ median (IQR)	Ever received biologic drug (%)	Ever received IV cyclophosphamide (%)
Total cohort (n = 379)^c^	40 (24.5–47.5)	4 (2.2–7.0)	77 (20)	89 (23)
Anti-TIF1γ (n = 68)	40 (25–47)**	5.2 (2.6–7.6)	23 (34)**	25 (37)*
Anti-NXP2 (n = 59)	29.5 (16–43)***	4.6 (2.7–6.9)	11 (19)	10 (17)
Anti-MDA5 (n = 23)	45 (38–52)***	4.1 (3.1–6.7)	1 (4)	6 (26)
Anti-PmScl (n = 20)	45.5 (22–49)	3 (1.5–6.9)	3 (15)	5 (25)
Anti- Mi2 (n = 15)	29 (15–38)***	4.8 (2.45–7)	1 (7)	3 (20)
Anti-U1RNP (n = 14)	46.5 (42–49)***	3.4 (1–5.2)	2 (14)	1 (7)
Anti-SRP (n = 7)	**26 (9.8**–42)***	6.6 (3.5–6.9)	2 (29)	2 (29)
Anti-synthetase^d^ (n = 6)	44.5 (41–48)	3.3 (2.4–5.5)	1 (17)	2 (33)
Anti-HMGCR (n = 4)	15 (1–30)***	6.6 (4.4–8.6)	4 (100)	2 (50)
Anti-SAE (n = 3)	39 (23–47)	3.5 (2–7)	0	0
Other^e^ (n = 5)	40 (25–47)**	4.1 (2.3–7)	2 (40)	1 (20)
Nil identified^f^ (n = 155)	43 (27–48)	3.2 (2.0–6.0)	28 (18)	32 (21)

Statistically significant associations are highlighted in bold.

*P < 0.05, **P < 0.01, ***P < 0.001, PM; polymyositis.

CMAS; Childhood Myositis Assessment Score, PGA; Physician Global Assessment of disease activity.

a Data available for 355 patients.

b Data available for 370 patients.

c Including 8 patients with other idiopathic inflammatory myopathy.

d 3 patients anti-Jo-1, 2 patients anti-PL12 and 1 patient anti-PL7.

e 1 anti-Ku, 1 anti-Scl70, 1 anti-Ro60, 1 anti-U3RNP, 1 anti-mitochondrial antibody.

f No known autoantibody identified using the techniques described (42% of this group have unidentified bands visible on immunoprecipitation).

### 3.4 Myositis specific autoantibodies

#### 3.4.1 Anti-transcription intermediary factor 1 γ (anti-TIF1 γ, anti-p155/140)

Anti-TIf1γ is the most prevalent autoantibody in the myositis cohort; identifiable in 18% of patients. It was associated with cutaneous ulceration (OR 3.08 (1.65–5.73), p < 0.001) and greater muscle weakness as determined by CMAS (OR 1.15 (1.07–1.24), p < 0.001). These patients were more likely to have received treatment with a biologic drug (OR 2.37 (1.30–4.31), p = 0.005) and/or intravenous cyclophosphamide (OR 2.09 (1.17–3.72), p = 0.013).

#### 3.4.2 Anti-nuclear matrix protein 2 (anti-NXP2, anti-MJ, anti-p140)

Anti-NXP2 was present in 15% of myositis patients and was associated with a younger age at disease onset (OR 0.91 (0.84–0.98), p = 0.012). It was also associated with greater muscle weakness (OR 1.44 (1.35–1.55), p < 0.001). We have previously published that this Ab is associated with risk of calcinosis [2]. In this larger cohort the association between anti-NXP2 and the development of calcinosis bordered on significance (OR 1.82 (0.99–3.36), p = 0.055).

#### 3.4.3 Anti-melanoma differentiation associated protein 5 (Anti-MDA5, anti-CADM 140)

Anti-MDA5 is the third most common autoantibody in the myositis cohort and was identified in 6% of patients. It was inversely associated with muscle weakness determined by CMAS (OR 0.57 (0.49–0.66), p < 0.001), confirming our previous observation that patients with anti-MDA5 are less likely to be weak [3]. Anti-MDA5 was also associated with cutaneous ulceration (OR 5.74 (2.32–14.17), p < 0.001), and arthritis (OR 2.81 (1.140–6.94), p = 0.023). Data on the presence or absence of interstitial lung disease in the cohort was incomplete and not included in regression analyses however, using the same cohort we have previously shown that 19% of those with anti-MDA5 developed interstitial lung disease and none rapidly progressive interstitial lung disease [3].

#### 3.4.4 Anti-Mi2

Anti-Mi2 was identified in 4% of myositis patients and was associated with greater muscle weakness (OR 1.70 (1.53–1.89), p < 0.001) dysphagia (OR 3.09 (1.05–9.06), p = 0.040), and oedema (OR 4.10 (1.26–13.30), p = 0.019) than other patients in the cohort.

#### 3.4.5 Anti-signal recognition peptide (anti-SRP)

**Fig. 4:**
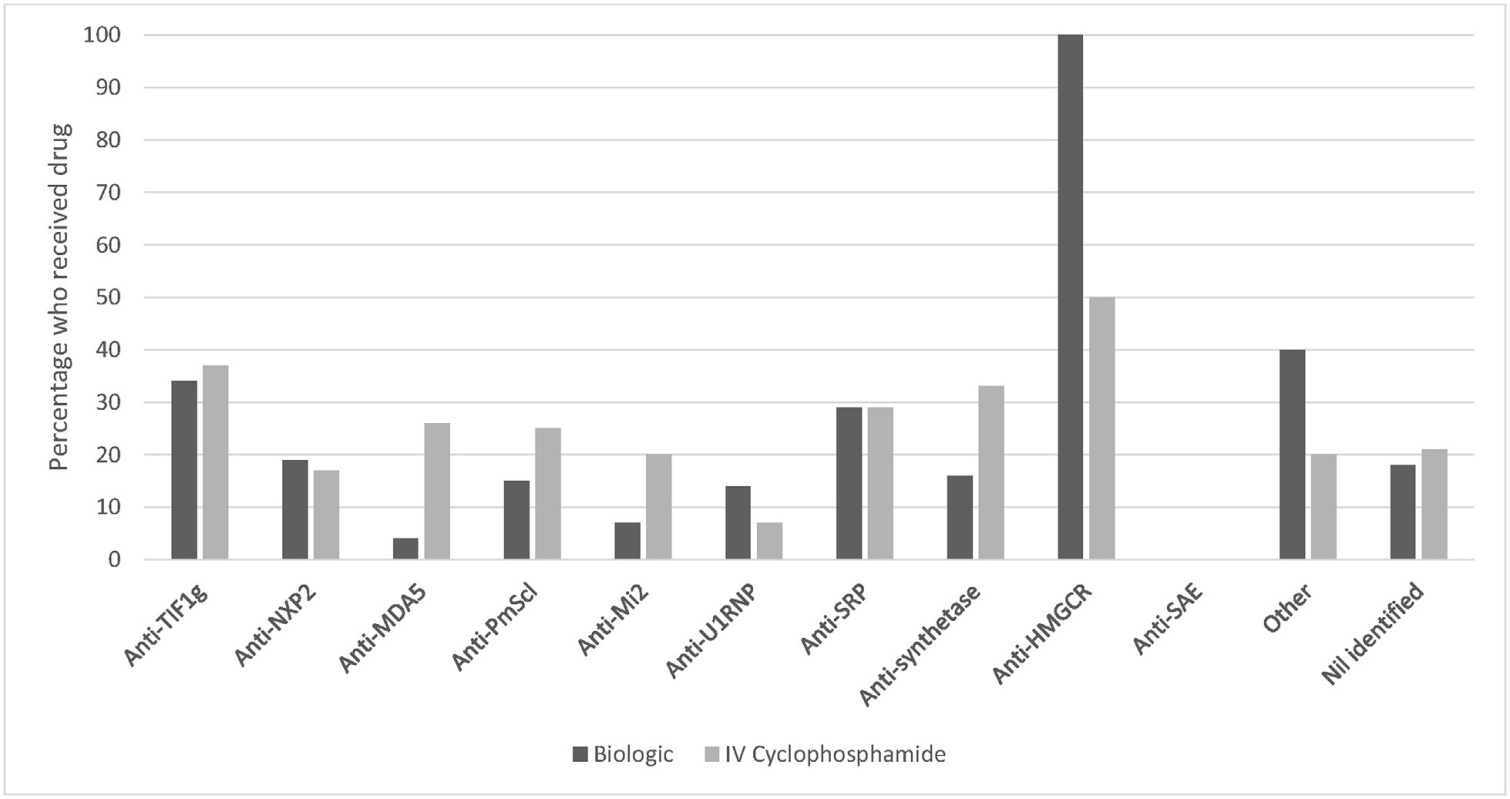
The proportion of myositis patients within each autoantibody defined subgroup who received treatment with intravenous cyclophosphamide (dark grey) and/or a biologic drug (light grey) is shown.

Anti-SRP was rare and found in just 2% of myositis patients. These patients were more likely to be classified as polymyositis (OR 66.6 (7.76–571.61), p < 0.001). Anti-SRP was associated with greater muscle weakness (OR 1.15 (1.07–1.24), p < 0.001) and cutaneous ulceration (OR 7.65 (1.65–35.54), p = 0.009). No statistically significant associations were seen with treatment received but a greater proportion of these patients did receive both intravenous cyclophosphamide and biologic drugs, see Fig. 4.

#### 3.4.6 Anti-tRNA synthetase

An anti-tRNA synthetase autoantibody was identified in 2% of myositis patients who were also more likely to be older at disease onset (OR 1.38 (1.08–1.76), p = 0.009). Anti-tRNA synthetase autoantibodies were inversely associated with muscle weakness (OR 0.77 (0.61–0.98), p = 0.041), suggesting patients are less likely to be weak. They were also associated with the development of lipoatrophy (OR 7.06 (1.13–43.96), p = 0.036). Three patients had anti-Jo-1 autoantibodies, two anti-PL12 and one anti-PL7. Those patients with anti-PL12 and anti-PL7 developed interstitial lung disease but this did not occur in the patients with anti-Jo-1.

#### 3.4.7 Anti-3-hydroxy-3-methyl-glutaryl-coenzyme A reductase (anti-HMGCR)

Anti-HMGCR was found in just four patients (1%). These patients were more likely to be classified as polymyositis (OR 165.50 (15.06–1840.69), p < 0.001), had greater muscle weakness (OR 2.33 (1.97–2.76), p < 0.001) and suffer from dysphagia (OR 10.48 (1.05–104.91), p = 0.046). Whilst no statistically significant association was seen with treatment received it is noteworthy that all four patients ultimately received biologic treatment and 50% IV cyclophosphamide, see Fig. 4.

#### 3.4.8 Anti-small ubiquitin-like modifier activating enzyme (anti-SAE)

Only three patients had anti-SAE autoantibodies. No specific phenotype associations were identified. In keeping with previous descriptions of the anti-SAE associated disease [25]two patients presented with JDM rash with very little or no muscle involvement but subsequently developed weakness and raised muscle enzymes. In both of these patients skin disease has been a persistent problem. In contrast the third patient presented following a seven month history of myalgia and weakness with no rash. Myositis was diagnosed on the basis of raised muscle enzymes plus consistent MRI and muscle biopsy findings. This patient developed typical cutaneous features of JDM two years later.

#### 3.4.9 Myositis associated autoantibodies

##### 3.4.9.1 Anti-polymyositis scleroderma (anti-PmScl)

Anti-PmScl was identified in 20 (5%) of myositis patients. No patient was classified as polymyositis or polymyositis overlap but 15 (75%) were classified as dermatomyositis overlap and, in keeping with the classic description of this autoantibody, nine patients (45%) were classified as overlap with scleroderma. The remaining 11 patients were described as dermatomyositis (five patients), mixed connective tissue disease (four patients), dermatomyositis polyarthritis overlap (one patient) or dermatomyositis lupus overlap (one patient). In addition to overlap disease anti-PmScl were also associated with the development of calcinosis (OR 3.31 (1.29–8.52), p = 0.013) and lipoatrophy (OR 3.08 (1.09–8.77), p = 0.035).

##### 3.4.9.2 Anti-U1 ribonucleoprotein (anti-U1RNP)

Anti-U1RNP were identified in 14 (4%) of myositis patients and were associated with an older age at disease onset (OR 1.19 (1.04–1.36), p = 0.011) and polymyositis or polymyositis overlap (OR 45.4 (6.88–299.79), p < 0.001). Patients were less likely to have a lower CMAS score (OR 0.54 (0.44–0.66), p < 0.001) suggesting patients are likely to be less weak. Seven (50%) of myositis patients were classified as having overlap disease or mixed connective tissue disease. Anti U1RNP was also detected in the JSLE patient cohort, confirming previous reports [26]. Phenotypic data was not examined for those patients with JSLE.

##### 3.4.9.3 Other autoantibodies

‘Other’ autoantibodies identified were anti-Ku, anti-Scl70, anti-Ro60, anti-U3RNP and anti-mitochondrial antibody. Collectively they were more likely to be identified in older patients (OR 1.46 (1.12–1.90), p = 0.005) and were associated with polymyositis or polymyositis overlap (OR 33.3 (2.58–429.81), p = 0.007).

##### 3.4.9.4 No identifiable autoantibodies

No clinical associations were identified with the absence of a detectable autoantibody.

## 4 Discussion

Juvenile myositis is a very rare disease and our large study makes a significant contribution to the available evidence on autoantibody associated disease phenotype. The study is also unique by the inclusion of several large non-myositis juvenile control groups tested for MSA/MAA, using the same methodology, in parallel with myositis patients. Consistent with previous studies the MAA anti-U1RNP was common in the group with JSLE [26], in addition to older patients with myositis-overlap disorders. In contrast MSA were exclusively found in juvenile-onset myositis and accordingly were 100% specific and 49% sensitive for identifying juvenile patients with myositis. The specificity is far superior to anti-nuclear antibodies which, whilst identifiable in over 70% of juvenile myositis patients [27], were also found in nearly 60% of the JIA patients and 90% of the JSLE patients [27]. Therefore, the identification of a MSA should be considered highly suggestive of the presence of myositis or an associated overlap disorder.

As expected in patients with juvenile-onset myositis the vast majority had dermatomyositis and the absence of cutaneous disease was rare. Those patients with polymyositis had either anti-SRP, anti-HMGCR, anti-U1RNP or ‘other’ MAA. This is in stark contrast to adult patients where anti-synthetase autoantibodies predominate and suggests that adult and juvenile-onset polymyositis are likely to present very differently [28]. This is important to recognise as the differential diagnosis of muscle weakness is wide and particularly for those children with anti-SRP and anti-HMGCR who often present with profound weakness, very elevated muscle enzymes and have a slow/poor response to treatment, the absence of a typical dermatomyositis rash can prompt ongoing investigation for other muscle disorders [29], [30].

The rarity of IIM combined with disease heterogeneity has hampered the development of good quality clinical trials and as reported in a Cochrane and other systematic literature reviews the evidence base for treatment remains very limited [31], [32], [33]. Despite limited evidence standard treatment for JIIM in the UK consists of immunosuppression with corticosteroids and methotrexate, a treatment regime which has recently been confirmed by a large international trial [2], [3], [4], [5], [6], [7], [8], [9], [10], [11], [12], [13], [14], [15], [16]. It is interesting to note that patients with some autoantibodies are more likely to receive additional ‘aggressive’ treatment with IV cyclophosphamide and/or biologic drugs, suggesting treatment resistance, severe disease or both. A significant association was seen between these more powerful treatments and anti-TIF1γ, the most common autoantibody in our cohort. Patients with anti-HMGCR, anti-SRP and anti-synthetase autoantibodies also received one or other of these treatments more often but it is likely this study was underpowered to demonstrate a significant relationship with these rarer autoantibody subgroups. While we acknowledge that treating physicians may have had some knowledge of autoantibody status through routine diagnostic testing this is typically limited in the UK, and traditional autoantibodies detected via standard methods form a very small proportion of our juvenile cohort. Autoantibody testing for this study was performed for research purposes only, on stored serum samples, in a designated university laboratory, often many years after diagnosis. Furthermore, the results were not fed back to the treating physician and it is therefore unlikely that autoantibody status per se could have influenced treatment choice.

Interestingly, it is not always those autoantibody categories associated with features of severe disease, for example greater muscle weakness as determined by CMAS, dysphagia and oedema in the case of anti-Mi2, and greater muscle weakness with anti-NXP2 that have a greater proportion of patients receiving aggressive therapy, suggesting some subgroups may be more treatment responsive. In the adult literature there is emerging evidence of a differential response to B-cell depletion based on autoantibody subgroup, suggesting that autoantibody subgroup does influence treatment response [34], [35]. To date however very few randomised controlled trials in adult or paediatric patients have determined autoantibody status or adjusted for this in their analyses [31]. Our results suggest that adjusting for autoantibody status will be important in future therapeutic clinical trials to prevent confounding. We know that early treatment is crucial for good outcomes in juvenile myositis and therefore selecting a successful treatment strategy from the outset is critical [9], [10], [11], [12]. In the future autoantibody testing may facilitate the earlier identification of those patients ultimately requiring a more aggressive treatment approach.

A significant strength of the study is the use of immunoprecipitation to determine autoantibody status, in both myositis and controls, as the sensitivity and specificity of other commercially available assays has yet to be validated and may be prone to false positives [36], [37]. We have shown for the first time the absence of MSA in patients with JIA or JSLE and our results support previous studies suggesting the absence of MSA in patients with genetic muscle diseases [13]. In the future a similar comparison with patients with other dermatological diagnoses such as psoriasis would also be valuable since some children with JDM who present with mainly rash maybe wrongly diagnosed with psoriasis early in disease. Limitations of our study include a lack of prospectively collected data on interstitial lung disease and mortality. Information on interstitial lung disease is however available for those patients with anti-MDA5 and anti-synthetase autoantibodies where associations have previously been described. Only two deaths are known to have occurred in the patients studied.

## 5 Conclusion

Autoantibodies can be identified in the majority of children with juvenile-onset myositis, and MSA are exclusively found in those with myositis. The presence of an MSA should suggest a diagnosis of myositis and their association with clinically important disease features makes them useful prognostic biomarkers. There is evidence of an existing differential treatment approach for some autoantibody subgroups which warrants further investigation and has important implications for the design of future clinical trials.

## Funding

Funding for the UK JDM Cohort and Biomarker study has been provided by generous grants from the Wellcome Trust UK [085860], Action Medical Research UK [SP4252], The Myositis Support Group UK,Arthritis Research UK [14518, 20164], The Henry Smith Charity and Great Ormond Street Children's Charity [V1268], and the National Institute for Health Research (NIHR) Translational Research Collaboration Rare Diseases (TRC-RD). This research was supported by the NIHR Biomedical Research Centre at Great Ormond Street Hospital for Children NHS Foundation Trust and Institute of Child Health University College London (UCL). The JDM Cohort study and the UK Juvenile Systemic Lupus Erythematosus (JSLE) Cohort Study and Repository are both adopted onto the NIHR Comprehensive Research Network.

ST and this project were funded by the BMA Doris Hillier grant 2012 and a fellowship from the Bath Institute of Rheumatic Diseases.

FM is supported by the NIHR Biomedical Research Centre at Great Ormond Street Hospital for Children NHS Foundation Trust and University College London. The support of the Muscular Dystrophy UK to the Dubowitz Neuromuscular Centre (grant 512315 and Centre grant) and of the MRC to the Neuromuscular Centres in London (UCL) and Newcastle for the Biobank is also gratefully acknowledged.

Wendy Thomson is supported by the NIHR Manchester Musculoskeletal Biomedical Research Unit at Central Manchester University Hospitals NHS Foundation Trust and by Arthritis Research UK Centre for Genetics and Genomics (grant 20385). We are grateful to Arthritis Research UK for supporting CAPS (grant 20542).

The UK Juvenile Systemic Lupus Erythematosus (JSLE) Cohort Study and Repository is supported in part by funding from LUPUS UK. Recruitment and sample collection of JSLE and healthy control patients was supported by the NIHR-funded Alder Hey Clinical Research Facility for Experimental Medicine, and the Arthritis Research UK (ARUK-20621), Alder Hey Charity and University of Liverpool-funded UK Experimental Arthritis Treatment Centre for Children.

## Conflicts of interest

None.
